# Non-technical health care quality and health system responsiveness in middle-income countries: a cross-sectional study in China, Ghana, India, Mexico, Russia, and South Africa

**DOI:** 10.7189/jogh.08.020417

**Published:** 2018-12

**Authors:** Pascal Geldsetzer, Annie Haakenstad, Erin Kinsella James, Rifat Atun

**Affiliations:** 1 Department of Global Health and Population, Harvard T.H. Chan School of Public Health, Boston, Massachusetts, USA; 2 Department of Global Health and Social Medicine, Harvard Medical School, Boston, Massachusetts, USA

## Abstract

**Background:**

While there is increasing recognition that the non-technical aspects of health care quality – particularly the inter-personal dimensions of care – are important components of health system performance, evidence from population-based studies on these outcomes in low- and middle-income countries is sparse. This study assesses these non-technical aspects of care using two measures: health system responsiveness (HSR), which quantifies the degree to which the health system meets the expectations of the population, and non-technical health care quality (QoC), for which we ‘filtered out’ these expectations. Pooling data from six large middle-income countries, this study therefore aimed to determine how HSR and QoC vary between countries and by individuals’ sociodemographic characteristics within countries.

**Methods:**

We pooled individual-level data, collected between 2007 and 2010, from nationally representative household surveys of (primarily) adults aged 50 years and older in China, Ghana, India, Mexico, Russia, and South Africa. The outcome measure was a binary indicator for a ‘bad’ rating (HSR: “very bad” or “bad” on a five-point Likert scale; QoC: a worse rating of one’s own visit than that of the character in an anchoring vignette) on at least one of seven dimensions for the most recent primary care visit.

**Results:**

23 749 adults who reported to have sought primary care during the preceding 12 months were includedin the analysis. The proportion of participants who gave a bad rating for their last primary care visit on at least one of seven dimensions varied from 4.3% (95% confidence interval (CI) = 2.8-6.7) in China to 33.1% (95% CI = 23.6-44.2) in South Africa for HSR, and from 17.0% (95% CI = 11.4-24.5) in Russia to 50.8% (95% CI = 46.0-55.6) in Ghana for QoC. There was a strong negative association between increasing household wealth and both bad HSR and QoC in India and South Africa.

**Conclusions:**

Achieving universal health coverage (UHC) with good-quality health services (“effective UHC”) will require efforts to improve HSR and QoC across the population in Ghana and South Africa. Additionally, a particular focus on raising HSR and QoC for the poorest population groups is needed in India and South Africa.

Universal health coverage (UHC) aims to ensure that everyone can access and use needed health services without risking financial ruin or impoverishment [1]. To date, the focus of UHC has largely been on i) raising the proportion of the population with access to health services, ii) increasing the range of available health services, and iii) reducing the proportion of the costs that users must bear through immediate payments. More recently, however, policy and research attention has begun to focus not just on affordable coverage of health services but also on ensuring *good quality* of provided health services in an effort to achieve “effective UHC” [2-4].

Quality of health care services can be broadly divided into technical and non-technical aspects of care (with the latter sometimes also being referred to as “inter-personal” or “non-clinical”) [5]. Health system responsiveness (HSR), which the World Health Organisation (WHO) considers to be one of the three fundamental goals of a health system [6], can be viewed as being a measure of the non-technical quality of health care [7]. The WHO has defined HSR as “the ability of the health system to meet the population's legitimate expectations regarding their interaction with the health system, apart from expectations for improvements in health or wealth” [6]. This definition suggests that – unlike an objective measure of the non-technical quality of care – HSR ratings should not be adjusted for respondents’ expectations. It is possible, therefore, for a health system that provides low non-technical quality of health care to achieve high HSR if the population’s expectations of HSR are low (and vice-versa). This study thus assessed HSR and non-technical quality of care as two distinct – yet related – concepts.

When measuring responsiveness of a health system, it is critically important to assess the *distribution* of HSR across the population in addition to the average level of HSR experienced by the population. According to WHO, “fairness means that [the health system] responds equally well to everyone, without discrimination or differences in how people are treated. The distribution of responsiveness matters, just as the distribution of health does” [6]. The first attempt to measure and report HSR across countries was for the WHO World Health Report 2000 [6]. However, this assessment consisted merely of key informant interviews in 35 countries, and the findings were used to impute HSR in other countries using country-level characteristics (eg, per capita income). More recently, however, the WHO has included questions on HSR in the first wave of its Study on global AGEing and adult health (SAGE): a population-based household survey undertaken among primarily older adults in China, Ghana, India, Mexico, Russia, and South Africa. This study pools SAGE data sets across these six countries. In line with the WHO’s emphasis on the need to assess both the mean level and “fairness” of HSR [6], our aim in this study was to determine i) the mean level of HSR and non-technical quality of care achieved by each of the six SAGE countries across their populations, and ii) differences in HSR and non-technical quality of care between population groups within these countries.

## METHODS

We pooled data from all six countries (China, Ghana, India, Mexico, Russia, and South Africa) included in the first wave of the SAGE surveys, carried out between 2007 and 2010 [8]. Reasons to survey these six countries in the SAGE study included a wish to achieve representation from different geographic regions of the world as well as to cover countries that are at different stages of economic development and have varying epidemiological profiles [8].

### Sampling and data collection

SAGE employed multistage cluster sampling to select participating households, which is described in detail elsewhere [8]. While the SAGE surveys aimed to be nationally representative among adults aged 50 years and older, they also included a smaller sample of adults aged 18 to 49 years. Among all selected adults, the percentage of adults who was aged 18-49 years at the time of the survey was 11% in China, 16% in Ghana, 42% in India, 16% in Mexico, 10% in Russia, and 9% in South Africa.

Data were collected via face-to-face interviews. Paper-based questionnaires were administered in Ghana, India, Russia, and South Africa. All interviews in Mexico and one half of the interviews in China were computer-assisted. Interviews lasted a mean of 2.5 hours [8], and included questionnaires, anthropometric measurements (height, weight, waist and hip circumference), blood pressure measurements, and physical tests (eg, grip strength and cognition). The following questionnaires were administered: i) a household questionnaire that included a household roster as well as questions on household wealth; ii) an individual questionnaire on health, well-being, and health care, including health system responsiveness; and iii) a verbal autopsy module. More details on the sampling methodology and data collection have been published elsewhere [8].

### Measuring health system responsiveness

All respondents were asked whether they sought outpatient care in the preceding 12 months, and/or inpatient care in the 36 months prior to the survey. Those who did were asked to rate the following aspects of their most recent outpatient and/or inpatient visit on a five-point Likert scale (ranging from “very good” to “very bad”) with the abbreviation of each domain, as used in this manuscript, shown in brackets: (1) wait time (‘wait’), (2) respectful communication by the health care provider (‘respect’), (3) clarity of information provided (‘communication’), (4) cleanliness of the health care facility (‘cleanliness’), (5) dignity of care (‘dignity’), (6) opportunity for the patient to be involved in making decisions about health care (‘shared decision-making’), and (7) freedom of choosing a health care provider (‘choice’). The questions and answer options are detailed in Appendix S1 in **Online Supplementary Document(Online Supplementary Document)**. Outpatient care was defined as any health care not including an overnight stay in a hospital or long-term care facility. Ratings of outpatient care were used for the primary analyses because the number of respondents who accessed inpatient care in the 36 months prior to the survey was small (eg, only 86 respondents in Mexico) and thus our power to detect differences in the inpatient care experience between countries, and population groups within each country, comparatively low.

The WHO’s definition of HSR (“the ability of the health system to meet the population's legitimate expectations regarding their interaction with the health system, apart from expectations for improvements in health or wealth” [6]) suggests that HSR can be appropriately summarised as a binary variable indicating whether expectations are met or not. Our dependent variable for HSR was thus a binary indicator for a “bad” HSR rating, which was defined as a rating for the last outpatient visit of “very bad” or “bad” on a five-point Likert scale (see Appendix S1 in **Online Supplementary Document(Online Supplementary Document)**). To allow for simple interpretability of the results, we chose to summarise HSR across the seven dimensions as a binary indicator for whether the respondent provided a bad rating on at least one HSR dimension for his/her last outpatient visit.

### Measuring non-technical quality of health care

Non-technical quality of care (henceforth abbreviated as QoC) was assessed using the same set of questions as for HSR except that respondents’ expectations were ‘filtered out’ using anchoring vignettes. Specifically, all respondents were presented with one vignette for each of the seven QoC domains. Each vignette described a scenario of care (see Appendix S2 in **Online Supplementary Document(Online Supplementary Document)** for the vignette texts). Respondents rated the experience of a hypothetical patient in this vignette on the same five-point Likert scale used to rate their own care. We used these vignette ratings to ascertain respondents’ expectations of QoC, and to then adjust respondents’ subjective ratings of their health care for their own expectations. To do so, we employed a non-parametric method developed by King et al. because it is a simple, intuitive approach that requires no additional assumptions [9,10]. In line with the definition of the outcome variable for HSR, we created an indicator variable for a bad rating of QoC, which was defined as assigning a lower score (on the same five-point Likert scale and separately for each dimension) to one’s own visit than to the visit described in the vignette scenario. Using vignettes to adjust responses for participants’ expectations relies on two assumptions: response consistency and vignette equivalence. Response consistency exists if respondents have the same expectations for the hypothetical vignette patients as for themselves. Vignette equivalence requires that all respondents interpret a given vignette in the same manner. In other words, the assumption of vignette equivalence is fulfilled if all respondents interpret the vignette to represent the same absolute level of QoC on a ‘true’ latent scale, and then merely apply their personal expectations to choose a response (eg, “very good” or “good”) to reflect this “true” latent scale. King et al. and Hopkins et al. provide a more in-depth discussion of these assumptions [10,11]. As for HSR, QoC was summarised across the seven dimensions as a binary indicator for whether the respondent provided a bad rating on at least one QoC dimension for his/her last outpatient visit. The vignettes were originally developed for, and used in, the World Health Surveys, which were implemented between 2002 and 2004 in 70 countries and had a total sample size of over 300 000 individuals [12].

### Independent variables

We used the following independent variables: health care provider type (private, public, and “other”, whereby ”other” referred to charity clinics, charity hospitals, home visits, “other”, and “don’t know”), household wealth quintile, educational attainment (ranging from no schooling to completing college or university), rural vs urban residence, country, age group (categorical), sex, and a binary variable for whether the household member had health insurance. For Mexico, there were no data to ascertain whether each household member had health insurance. Instead, we used a binary indicator in the Mexico data set for whether the costs of the last outpatient care visit were covered by a health insurance. Household wealth was assessed through a country-specific list of 20-25 household assets, which were summarised in a household wealth index using principal component analysis, as per the methodology developed by Filmer and Pritchett [13].

### Statistical analysis

To assess the absolute level of each outcome variable in a country and compare it between countries, we calculated and then plotted the mean probability of a bad rating on at least one dimension for each country, while accounting for the complex survey design with sampling weights (using the srvyr R package [14]). We used a Wald test (following an F-distribution) for testing the joint significance of ‘country’ as a categorical independent variable in a logistic regression model for survey-weighted data to determine whether the mean level of a bad HSR and QoC rating differed significantly between the study countries. Because the length of the recall period (ie, the time between the interview and the last outpatient care visit within the preceding 12 months) might have systematically impacted on a respondent’s rating, we also produced this plot among only those respondents whose last outpatient care visit was less than or equal to two months prior to the interview.

To study how HSR and QoC varied within countries between different population groups, we used regressions with a country-level fixed effect adjusting standard errors for clustering at the level of the primary sampling unit (PSU – a village, neighbourhood, or census enumeration area, depending on the country and setting) [15]. The binary outcomes modelled in these regressions were relatively common, and thus the Odds Ratio will differ substantially from the more easily interpretable Risk Ratio (RR). We therefore modelled our data using Poisson regression models with a robust error structure [16], which yields a RR. Specifically, including a country-level fixed effect in each model, we regressed the outcome i) separately onto each independent variable, ii) onto all socio-demographic variables (ie, household wealth quintile, education, rural vs urban residency, age group, and sex), and iii) onto all independent variables. The p-values testing for statistical significance of each RR were adjusted for multiple hypothesis testing using both the Holm method (which controls the family-wise error rate without any assumptions) and the Benjamini-Hochberg method (which controls the false discovery rate) [17,18]. The multiple hypothesis adjustment was performed separately for each of the two outcome variables, and separately for each of the three types of regression models outlined above. In our regression table, we thus show for each RR the p-value when unadjusted for multiple hypothesis testing, when adjusted using the Holm method, and when adjusted using the Benjamini-Hochberg method. As a sensitivity analysis, we show in Figure S1 and Table S1in **Online Supplementary Document(Online Supplementary Document)** the results of the same regressions when restricting the sample to those whose last outpatient care visit was less than or equal to two months prior to the survey.

Because we observed a large and significant correlation between household wealth quintile and the outcomes, we decided to explore how household wealth quintile was associated with the outcomes in each country and on each of the seven HSR dimensions. This decision was not made according to a pre-registered analysis protocol, and should therefore be interpreted as being exploratory in nature only. Nonetheless, we adjusted the p-values in these analyses for multiple hypothesis testing using the Holm method, which is more conservative than the Benjamini-Hochberg method [18]. We assessed the association between household wealth quintile and the outcomes by plotting the predicted probability of each outcome by household wealth quintile and country. These predicted probabilities – holding other co-variates at their observed values [“average marginal effects”] as recommended by Hanmer et al [19] – were obtained from multivariable logistic regressions (adjusting standard errors for clustering at the PSU-level) that were run separately for each country and included the following co-variates: household wealth quintile (categorical), age (continuous), sex (binary), rural or urban (binary), and health care provider type (categorical). The association between household wealth quintile and a bad rating on each HSR dimension was assessed by plotting the RR comparing the poorest to the richest household wealth quintile by country and HSR/QoC dimension.

Lastly, having observed important differences in HSR and QoC by household wealth quintile, we investigated whether health care provider type (public, private, or other) might be a mediator of the association between household wealth and these outcomes. This analysis was again not pre-specified and thus only exploratory.

Prior to data analysis, missing values were imputed using “iterative random forests” as implemented through the “missForest” R package [20,21]. All statistical analyses were run in R version 3.3.2 [22].

## RESULTS

### Sample characteristics

Individual response rates varied between countries: 93% in China, 81% in Ghana, 68% in India, 53% in Mexico, 83% in Russia, and 75% in South Africa. The SAGE team identified a shorter data collection period, and thus less time for multiple revisits of households, as an important reason for the low response rate in Mexico [8]. Across the six countries, 23 749 participants reported to have sought outpatient care in the 12 months prior to the survey (**Table 1**). The proportion of all respondents who sought outpatient care in the last 12 months varied from 18.4% in Mexico to 69.3% in India. A far smaller number (4120 respondents) sought inpatient care in the 36 months prior to the survey. India was the only country, in which the last outpatient visit was more likely to be from a private than a public provider (60.6% vs 23.1%, respectively). Russia had the lowest proportion (3.7%) of respondents reporting to have sought their last outpatient care from a private provider.

**Table 1:** Unweighted sample characteristics by country

	China	Ghana	India	Mexico	Russia	South Africa
No.	15 050	5573	12 198	5448	4947	4227
Sought outpatient care in last 12 months, n (%)	6722 (44.7)	2967 (53.2)	8458 (69.3)	1001 (18.4)	2593 (52.4)	2008 (47.5)
-from a public provider, n (%)*	4543 (67.6)	1562 (52.6)	1957 (23.1)	650 (64.9)	2142 (82.6)	1450 (65.8)
-from a private provider, n (%)*	1834 (27.3)	476 (16.0)	5125 (60.6)	324 (32.4)	96 (3.7)	526 (33.3)
-from another provider, n (%)*,†	343 (5.1)	929 (31.3)	1374 (16.2)	27 (2.7)	354 (13.7)	32 (0.9)
*-Missing*, n (%)*	*2 (0.0)*	*0 (0.0)*	*2 (0.0)*	*0 (0.0)*	*1 (0.0)*	*0 (0.0)*
Sought inpatient care in last 36 months, n (%)	1574 (10.5)	375 (6.7)	1039 (8.5)	86 (1.6)	747 (15.1)	299 (7.1)
-from a public provider, n (%)‡	1471 (93.5)	232 (61.9)	390 (37.5)	63 (73.3)	729 (97.6)	206 (68.9)
-from a private provider, n (%)‡	86 (5.5)	89 (23.7)	610 (58.7)	22 (25.6)	10 (1.3)	65 (2.2)
-from another provider, n (%)†,‡	12 (0.8)	52 (13.9)	39 (3.8)	1 (1.2)	8 (1.1)	2 (0.7)
*Missing*, n (%)‡	*5 (0.3)*	*2 (0.5)*	*0 (0.0)*	*0 (0.0)*	*0 (0.0)*	*26 (8.7)*
**Sample characteristics among those who sought outpatient care in the last 12 months:**							
Bad HSR rating§ (%)	4.3	24.1	11.2	13.9	14.5	30.7
*-Missing* (%)	2.3	2.1	0.8	0.0	17.2	2.2
Bad QoC rating (%)‖	18.7	52.9	27.3	25.5	20.7	26.9
*Missing* (%)	2.8	2.4	1.1	0.0	20.6	3.4
Mean age (SD)	61.1 (11.6)	61.3 (14.4)	50.4 (16.6)	63.6 (13.8)	63.5 (12.9)	61.4 (11.8)
*-Missing* (%)	*0.0*	*0.1*	*0.0*	*0.5*	*0.0*	*0.0*
**Age group (years, %):**							
<50	10.0	14.6	41.1	15.0	8.5	7.4
50-59	37.4	30.5	25.7	16.0	30.7	38.1
60-69	27.3	23.8	20.2	35.8	25.2	30.5
≥70	25.3	31.2	13.0	33.2	35.6	24.0
Female (%)	56.1	51.8	62.6	64.8	69.5	60.9
*-Missing* (%)	*0.0*	*0.0*	*0.0*	*0.0*	*0.0*	*0.0*
Rural (%)	53.4	56.0	73.7	28.3	23.6	30.6
*-Missing* (%)	*0.0*	*0.0*	*0.0*	*0.0*	*0.0*	*0.1*
**Wealth quintile:**							
-1 (poorest)	16.5	15.3	17.1	19.7	16.0	14.7
-2	18.1	18.4	19.2	19.0	20.0	17.8
-3	19.5	21.0	19.6	17.9	20.4	20.7
-4	22.9	22.2	21.1	22.1	21.3	24.6
-5 (wealthiest)	23.0	23.1	23.0	21.2	22.3	22.2
*-Missing* (%)	0.5	0.2	0.2	0.2	0.0	0.5
**Education (%):**							
No schooling	23.1	48.3	45.7	17.5	1.0	22.0
Some primary school	17.8	11.4	10.2	36.1	1.9	27.0
Completed primary school	18.3	12.5	15.8	23.0	7.1	24.4
Completed secondary school	21.2	6.0	11.8	10.6	19.3	14.0
Completed high school	13.9	17.9	10.5	3.2	51.0	7.2
Completed college or university	5.7	3.8	5.9	9.7	19.8	5.4
*-Missing* (%)	*0.0*	*0.4*	*0.0*	*0.0*	*0.1*	*16.6*
Has health insurance (%)	90.3	43.3	4.6	NA^¶^	99.8	19.0
*-Missing* (%)	*0.5*	*0.0*	*0.0*	*NA*^¶^	*0.3*	*0.6*

SD – standard deviation, NA – not applicable, HSR – health system responsiveness, QoC – quality of care

*The denominator for the percentage is the number of participants who sought outpatient care in the last 12 months.

†This includes charity clinics and hospitals, home visits, “other”, and “don’t know”.

‡The denominator for the percentage is the number of participants who sought inpatient care in the last 12 months.

§This is the percentage of respondents who provided a bad HSR rating (selecting “very bad” or “bad” on a 5-point Likert scale) on at least one of seven HSR dimensions.

‖This is the percentage of respondents who assigned a lower rating to their own visit than to the visit described in the vignette scenario on at least one of seven QoC dimensions.

¶Whether each household member has health insurance was not included in the Mexico data set available in the public domain.

Among those who sought outpatient care in the preceding 12 months (the primary analytical sample for this study), the mean age of participants was similar across the countries with the exception of India where additional women aged 18-49 years were recruited as part of a nested sub-study [8]. Across all countries, those who reported to have sought outpatient care in the previous 12 months were more likely to be from a richer (quintiles 4 or 5) than a poorer wealth quintile (quintiles 1 or 2). Educational attainment of the outpatient sample varied substantially between countries with respondents in Russia being the most educated (51.0% completed high school and 19.8% college or university). Ghana and India had the highest percentage of participants without any schooling (48.3% and 45.7%, respectively). The percentage of respondents with health insurance among the outpatient sample varied from 4.6% in India to over 90% in China (90.3%) and Russia (99.8%).

Less than four percent of observations for the dependent variables were missing in each country. The exception is Russia where 17.2% and 20.6% of observations were missing for a bad HSR and QoC rating, respectively. Regarding the independent variables, the percentage of observations that was missing was negligible (<1%) for all countries and variables with the exception of educational attainment among the outpatient sample in South Africa (16.6% missing) and the health care provider type for the last inpatient care visit in South Africa (8.7% missing).

### Differences between countries

**Figure 1:**
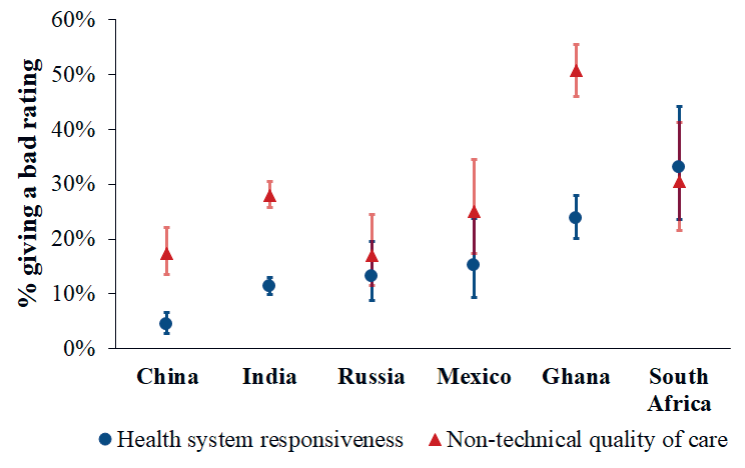
Percentage of respondents giving a bad rating for their last outpatient care visit, by country. For health system responsiveness, a ‘bad’ rating was a rating of “very bad” or “bad” on a five-point Likert scale. For non-technical quality of care, a “bad” rating was a rating of one’s experience for the most recent outpatient visit worse than that described in the vignette scenario. Vertical lines show 95% confidence intervals. Using a Wald test (that follows an F-distribution) for testing the joint significance of ‘country’ as a categorical independent variable in a logistic regression model for survey-weighted data, we rejected (at α<0.05) the null hypothesis that the mean probability of a bad outpatient rating is equal between countries with *P* < 0.001 for both outcomes.

**Figure 1** compares the six SAGE countries in their mean probability of a bad HSR and QoC rating for a respondent’s last outpatient visit. The p-value testing the null hypothesis that the mean probability of each outcome was equal between countries was <0.001. There was thus significant variation between countries in these outcome variables. Respondents in South Africa and Ghana were most likely to report a bad HSR experience (33.1%, 95% CI = 23.6-44.2, and 23.8%, 95% CI = 20.2-27.9], respectively) and those in China the least likely (4.3%, 95% CI = 2.8-6.7). For QoC, Ghana was the country with the highest probability of a bad rating with approximately half (50.8%, 95% CI = 46.0-55.6) of respondents reporting an experience worse than the vignette character’s on at least one QoC dimension. While the absolute difference in the country-level mean probability of a bad HSR and a bad QoC rating was highest for Ghana (HSR: 23.8%, 95% CI = 20.2-27.9; QoC = 50.8%, 95% CI = 46.0-55.6, the relative difference was highest for China (HSR = 4.3%, 95% CI = 2.8-6.7; QoC = 17.4%, 95% CI = 13.5-22.1) followed by India (HSR = 11.3%, 95% CI = 9.8-12.9; QoC = 2.8%, 95% CI = 25.7-30.5). The pattern between countries in the outcome variables was similar when restricting the sample to those 14 103 respondents for whom the last outpatient visit was less than, or equal to, two months prior to the survey (Figure S1 in **Online Supplementary Document(Online Supplementary Document)**). The pattern was also similar when examining the last inpatient, rather than outpatient, visit (Figure S2 in **Online Supplementary Document(Online Supplementary Document)**) with the exception of Mexico, which had a sample size of only 86 respondents for inpatient care questions.

### Differences between population groups within countries

Increasing household wealth quintile was negatively associated with both outcome variables in all regression models. While there was no clear trend between education and a bad HSR rating, a bad QoC rating was negatively associated with higher educational attainment in the model that only included education and country-level fixed effects as independent variables. The education trend with QoC was similar in the other regression models but most indicator variables for education did not reach significance. Age group, sex, and having health insurance were not significantly associated with the outcomes in any of the regressions. Residing in a rural rather than an urban area was associated with a higher risk of a bad QoC rating. For HSR, the association with rural residency was in the opposite direction but none of the RRs reached significance. Having had the last outpatient care visit with a public rather than a private provider was associated with a higher risk of a bad rating on at least one HSR dimension (RR = 2.17, 95% CI = 1.93-2.43, in model 7; RR = 2.14, 95% CI = 1.91-2.40, in model 9). However, for the QoC outcome, the RRs for health care provider type were close to one and insignificant. The regression results were similar, albeit with wider CIs due to the smaller sample size, when restricting the sample to those whose last outpatient care visit was not more than two months prior to the interview (Table S1 in **Online Supplementary Document(Online Supplementary Document)**).

**Figure 2:**
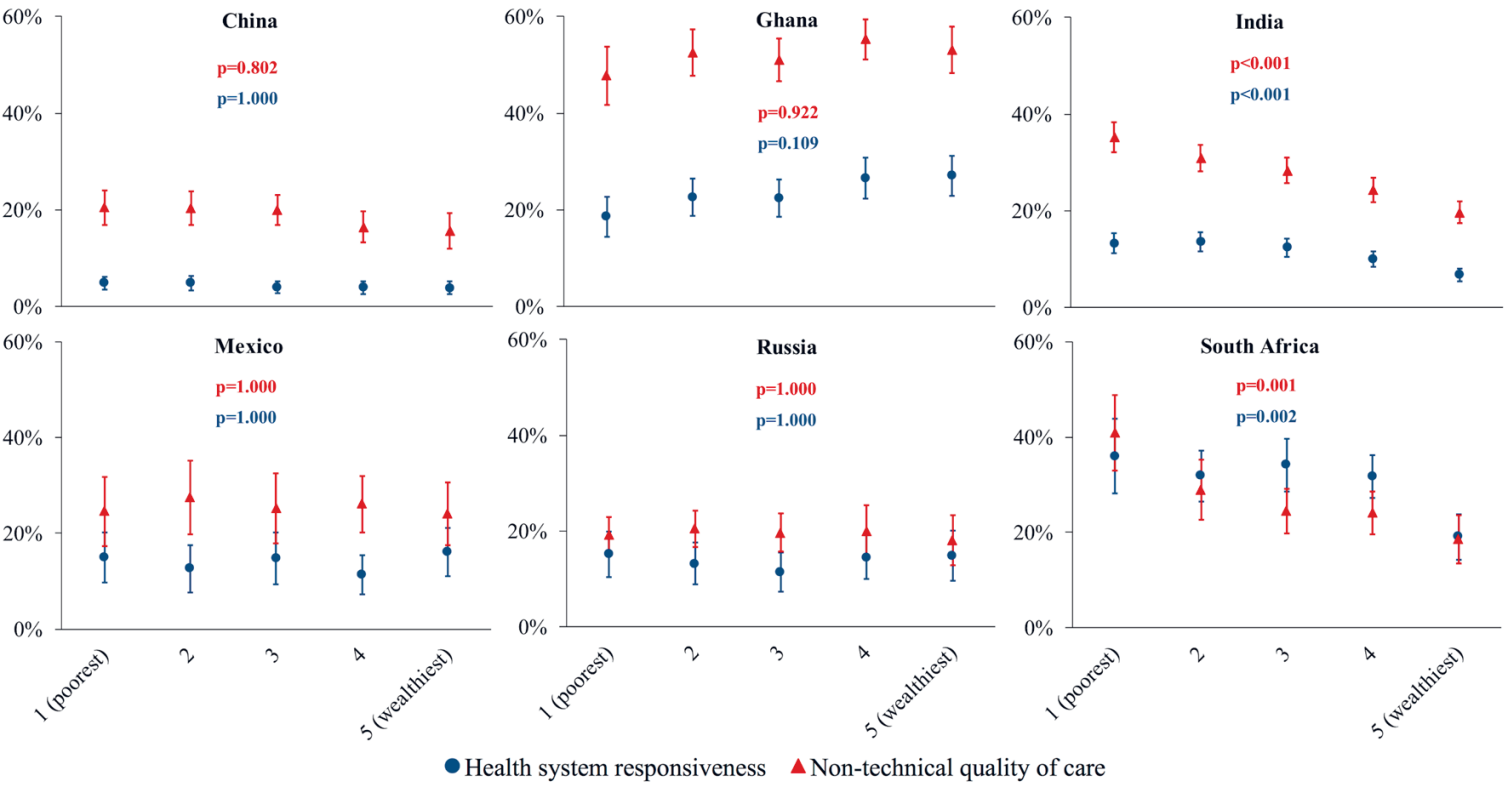
Predicted probability (y axis) of a bad outpatient care rating on at least one dimension, by wealth quintile and country. For health system responsiveness, a “bad” rating was a rating of “very bad” or “bad” on a five-point Likert scale. For non-technical quality of care, a ‘bad’ rating was a rating of one’s experience for the most recent outpatient visit worse than that described in the vignette scenario. Predicted probabilities were obtained from multivariable logistic regressions, run separately for each country, with the following co-variates: age (continuous), sex (binary), rural or urban (binary), wealth quintile (categorical), and health care provider type (categorical). The predicted probabilities for each wealth quintile were calculated holding other co-variates at their observed values (‘average marginal effects’) as recommended by Hanmer et al [19]. Vertical lines show 95% confidence intervals obtained through the delta method. The *P*-values shown are p-values for a Wald test testing the null hypothesis that the coefficients for each wealth quintile indicator variable are simultaneously equal to zero. These p-values were adjusted for multiple hypothesis (six hypotheses for each the HSR and QoC outcome) testing using the Holm method [17].

**Figure 3:**
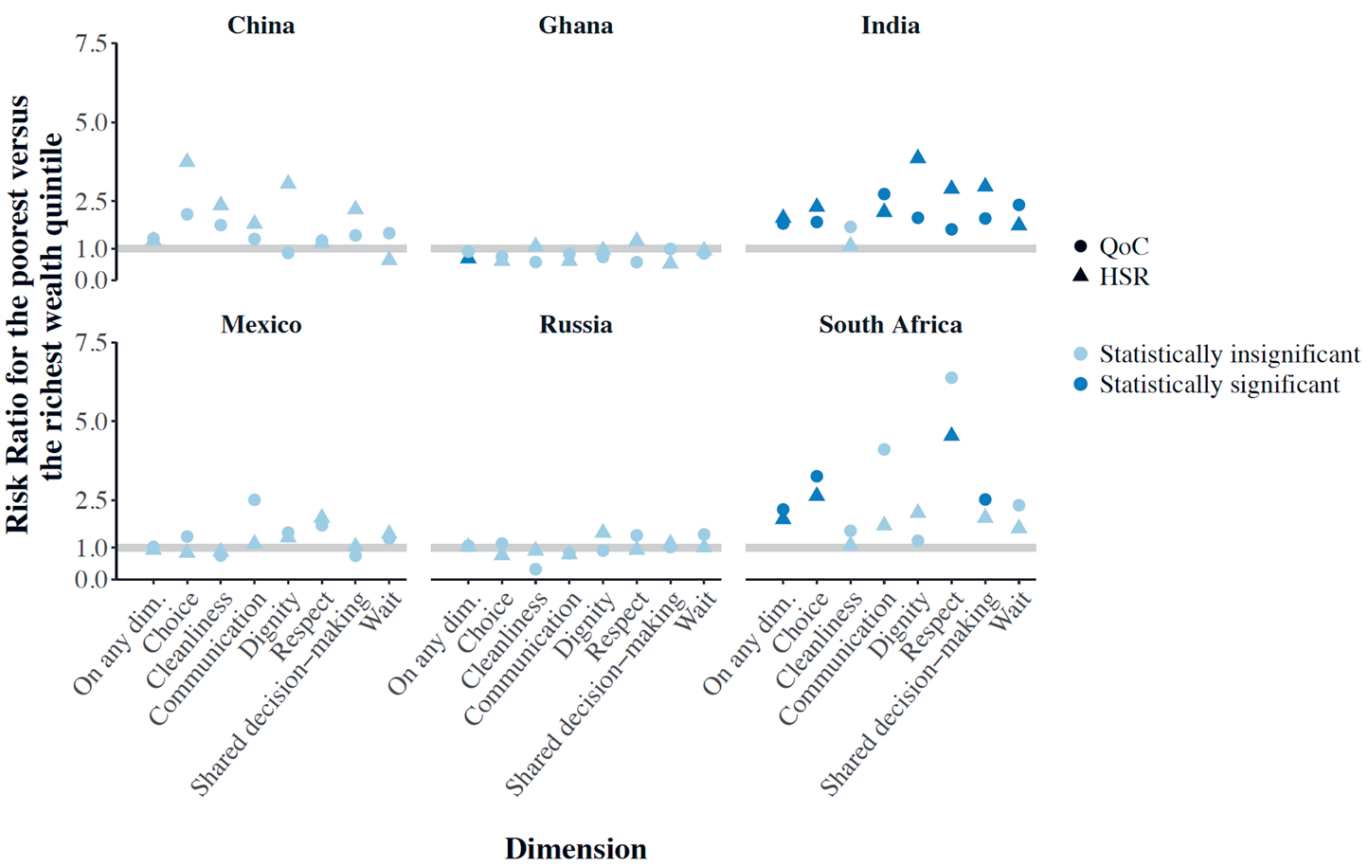
Risk of a bad outpatient care rating for the poorest vs the richest wealth quintile, by dimension and country. QoC – Non-technical quality of care, HSR – health system responsiveness, dim – dimension. Risk Ratios above 1.0 indicate that those in the poorest wealth quintile had a higher probability of reporting a bad experience than those in the wealthiest quintile. Risk Ratios were obtained from multivariable Poisson regressions with a robust error structure run separately for each country. The co-variates included in these regressions were age (continuous), sex (binary), rural or urban (binary), wealth quintile (categorical), health care provider type (categorical), and whether the household member had health insurance (binary). The *P*-values indicating statistical significance were adjusted – separately for each country – for testing seven hypotheses at once (one hypothesis for each dimension) using the Holm method [17]. *P*-values for “On any dimension” were not adjusted for multiple hypothesis testing. Standard errors were adjusted for clustering at the level of the primary sampling unit. “On any dim.” is the Risk Ratio for rating one’s last visit as ‘bad’ on at least one of the seven dimensions. For health system responsiveness, a ‘bad’ rating was a rating of “very bad” or “bad” on a five-point Likert scale. For non-technical quality of care, a ‘bad’ rating was a rating of one’s experience for the most recent outpatient visit worse than that described in the vignette scenario.

Disaggregating the relationship between the outcomes and household wealth quintile by country shows that the overall negative association between wealth and a bad HSR and QoC rating shown in **Table 2** is driven mainly by strong negative correlations in India and South Africa (**Figure 2**). **Figure 3** disaggregates the relative risk of a bad HSR and QoC rating between the least and most wealthy quintile by dimension. For both outcomes, those in the poorest household wealth quintile in India had a higher risk of giving a bad rating on all dimensions, except facility cleanliness, than those in the richest quintile. The differences in the risk of a bad HSR and QoC rating between these two wealth quintiles were not significant for any dimensions in the other countries except in South Africa for freedom of choosing a health care provider, respectful communication (for HSR), and shared decision-making (for QoC).

**Table 2:** Regressions of each outcome variable onto respondents’ characteristics, health care provider type, and country-level fixed effects (n = 23 749)*,†,‡

	Models 1-7				Model 8				Model 9			
	**RR**	***P-*value**	**P^Holm^**	**P^BH^**	**RR**	***P-*value**	**P^Holm^**	**P^BH^**	**RR**	***P-*value**	**P^Holm^**	**P^BH^**
***Outcome: bad health system responsiveness rating***§												
**Household wealth quintile:**												
1 (poorest)	1.00 (Ref)	–	–	–	1.00 (Ref)	–	–	–	1.00 (Ref)	–	–	–
2	0.99 (0.89-1.12)	0.919	1.000	0.976	0.99 (0.88-1.11)	0.875	1.000	0.966	1.00 (0.89-1.12)	0.963	1.000	0.967
3	0.94 (0.83-1.05)	0.280	1.000	0.433	0.93 (0.82-1.05)	0.225	1.000	0.507	0.94 (0.83-1.05)	0.270	1.000	0.417
4	0.90 (0.80-1.02)	0.098	1.000	0.278	0.89 (0.79-1.01)	0.060	0.725	0.282	0.89 (0.79-1.01)	0.067	0.939	0.241
5 (wealthiest)	0.73 (0.64-0.84)	<0.001	<0.001	<0.001	0.71 (0.62-0.82)	<0.001	<0.001	<0.001	0.74 (0.65-0.85)	<0.001	<0.001	<0.001
**Education:**												
No schooling	1.00 (Ref)	–	–	–	1.00 (Ref)	–	–	–	1.00 (Ref)	–	–	–
Some primary school	0.99 (0.89-1.10)	0.855	1.000	0.969	1.00 (0.90-1.12)	0.936	1.000	0.966	1.00 (0.90-1.11)	0.967	1.000	0.967
Completed primary school	0.98 (0.88-1.10)	0.737	1.000	0.919	1.01 (0.90-1.13)	0.880	1.000	0.966	1.02 (0.92-1.14)	0.678	1.000	0.844
Completed secondary school	0.81 (0.71-0.92)	0.002	0.023	0.007	0.85 (0.74-0.98)	0.022	0.282	0.152	0.88 (0.77-1.01)	0.074	0.963	0.241
Completed high school	0.97 (0.85-1.09)	0.576	1.000	0.816	1.04 (0.91-1.19)	0.557	1.000	0.867	1.08 (0.95-1.24)	0.229	1.000	0.388
Completed college or university	0.86 (0.71-1.05)	0.133	1.000	0.323	0.98 (0.81-1.19)	0.838	1.000	0.966	1.06 (0.88-1.28)	0.548	1.000	0.776
Rural	0.98 (0.88-1.10)	0.757	1.000	0.919	0.91 (0.81-1.02)	0.100	1.000	0.351	0.91 (0.81-1.01)	0.085	1.000	0.241
**Age group (years):**												
<50	1.00 (Ref)	–	–	–	1.00 (Ref)	–	–	–	1.00 (Ref)	–	–	–
50-59	0.95 (0.85-1.05)	0.277	1.000	0.433	0.95 (0.86-1.06)	0.360	1.000	0.630	0.94 (0.85-1.04)	0.204	1.000	0.386
60-69	1.00 (0.91-1.11)	0.979	1.000	0.979	1.00 (0.91-1.11)	0.966	1.000	0.966	0.98 (0.89-1.09)	0.727	1.000	0.844
≥70	0.93 (0.82-1.04)	0.191	1.000	0.407	0.91 (0.81-1.03)	0.150	1.000	0.421	0.90 (0.80-1.02)	0.102	1.000	0.247
Female	1.06 (0.99-1.14)	0.083	1.000	0.278	1.04 (0.97-1.11)	0.254	1.000	0.507	1.04 (0.98-1.12)	0.204	1.000	0.386
Has health insurance	0.93 (0.82-1.05)	0.234	1.000	0.433	–	–	–	–	0.98 (0.86-1.12)	0.745	1.000	0.844
**Provider type:**												
Private	1.00 (Ref)	–	–	–	–	–	–	–	1.00 (Ref)	–	–	–
Public	2.17 (1.93-2.43)	<0.001	<0.001	<0.001	–	–	–	–	2.14 (1.91-2.40)	<0.001	<0.001	<0.001
Other^6^	1.58 (1.37-1.83)	<0.001	<0.001	<0.001	–	–	–	–	1.54 (1.33-1.78)	<0.001	<0.001	<0.001
***Outcome: bad non-technical quality of care rating‖***												
**Household wealth quintile:**												
1 (poorest)	1.00 (Ref)	–	–	–	1.00 (Ref)	–	–	–	1.00 (Ref)	–	–	–
2	0.94 (0.88-1.01)	0.071	0.497	0.110	0.95 (0.89-1.02)	0.156	1.000	0.273	0.95 (0.89-1.02)	0.144	1.000	0.273
3	0.86 (0.80-0.93)	<0.001	0.001	<0.001	0.89 (0.83-0.95)	0.001	0.015	0.006	0.89 (0.83-0.95)	0.001	0.014	0.005
4	0.79 (0.73-0.86)	<0.001	<0.001	<0.001	0.83 (0.77-0.90)	<0.001	<0.001	<0.001	0.82 (0.76-0.89)	<0.001	<0.001	<0.001
5 (wealthiest)	0.67 (0.61-0.73)	<0.001	<0.001	<0.001	0.72 (0.66-0.80)	<0.001	<0.001	<0.001	0.72 (0.66-0.79)	<0.001	<0.001	<0.001
**Education:**												
No schooling	1.00 (Ref)	–	–	–	1.00 (Ref)	–	–	–	1.00 (Ref)	–	–	–
Some primary school	0.94 (0.88-1.01)	0.093	0.558	0.132	0.96 (0.90-1.03)	0.300	1.000	0.424	0.97 (0.90-1.04)	0.336	1.000	0.476
Completed primary school	0.92 (0.85-0.99)	0.020	0.198	0.042	0.97 (0.90-1.04)	0.421	1.000	0.491	0.98 (0.91-1.05)	0.526	1.000	0.596
Completed secondary school	0.83 (0.76-0.90)	<0.001	<0.001	<0.001	0.92 (0.84-1.00)	0.049	0.399	0.098	0.92 (0.84-1.00)	0.064	0.728	0.144
Completed high school	0.79 (0.73-0.86)	<0.001	<0.001	<0.001	0.90 (0.82-0.98)	0.014	0.154	0.049	0.90 (0.83-0.98)	0.019	0.246	0.064
Completed college or university	0.72 (0.64-0.82)	<0.001	<0.001	<0.001	0.88 (0.77-1.00)	0.044	0.399	0.098	0.88 (0.77-1.01)	0.061	0.728	0.144
Rural	1.22 (1.12-1.34)	<0.001	<0.001	<0.001	1.11 (1.02-1.21)	0.019	0.194	0.054	1.11 (1.02-1.22)	0.015	0.210	0.064
**Age group (years):**												
<50	1.00 (Ref)	–	–	–	1.00 (Ref)	–	–	–	1.00 (Ref)	–	–	–
50-59	1.01 (0.94-1.08)	0.774	1.000	0.877	1.01 (0.95-1.08)	0.708	1.000	0.709	1.02 (0.95-1.09)	0.653	1.000	0.694
60-69	1.05 (0.98-1.13)	0.133	0.663	0.173	1.04 (0.97-1.11)	0.303	1.000	0.424	1.04 (0.97-1.11)	0.291	1.000	0.450
≥70	1.03 (0.95-1.10)	0.510	1.000	0.619	0.99 (0.91-1.06)	0.709	1.000	0.709	0.99 (0.92-1.07)	0.771	1.000	0.771
Female	1.00 (0.95-1.05)	0.853	1.000	0.906	0.98 (0.93-1.03)	0.395	1.000	0.491	0.98 (0.93-1.03)	0.377	1.000	0.493
Has health insurance	0.92 (0.86-0.99)	0.035	0.312	0.065	–	–	–	–	0.97 (0.90-1.05)	0.446	1.000	0.541
**Provider type:**												
Private	1.00 (Ref)	–	–	–	–	–	–	–	1.00 (Ref)	–	–	–
Public	1.08 (1.00-1.18)	0.055	0.440	0.093	–	–	–	–	1.08 (0.99-1.16)	0.068	0.728	0.144
Other¶	1.00 (0.92-1.09)	0.941	1.000	0.941	–	–	–	–	0.95 (0.87-1.03)	0.198	1.000	0.337

RR – risk ratio, CI - confidence interval, Ref – reference level

*These regression models were Poisson regressions with a robust error structure [16]. Standard errors were adjusted for clustering at the level of the primary sampling unit.

†Models 1-7 included each of the independent variables shown in the table separately plus country-level fixed effects. Model 8 included household wealth quintile, education, rural vs urban, age group, sex, and country-level fixed effects as independent variables. Model 9 included household wealth quintile, education, rural vs urban, age group, sex, health insurance status, health care provider type, and country-level fixed effects as independent variables.

‡P^Holm^ and P^BH^ refer to *P*-values that were adjusted for multiple hypothesis testing using the Holm method and the method developed by Benjamini and Hochberg, respectively.[17, 18] Adjustment for multiple hypothesis testing was done separately for each of the two outcomes (HSR and QoC). *P*-values from models 1-7 were adjusted jointly (ie, for 17 hypotheses), while p-values from models 8 and 9 were adjusted separately (ie, 14 hypotheses and 17 hypotheses, respectively).

§A “bad” rating was a rating of “very bad” or “bad” (on a 5-point Likert scale) on at least one of seven health system responsiveness dimensions.

‖A “bad” rating was a rating of one’s experience for the most recent outpatient visit worse than that of the vignette character on at least one of seven non-technical quality of care dimensions.

¶This includes charity clinics and hospitals, home visits, “other”, and “don’t know”.

### Healthcare provider type as a possible mediator

**Figure 4:**
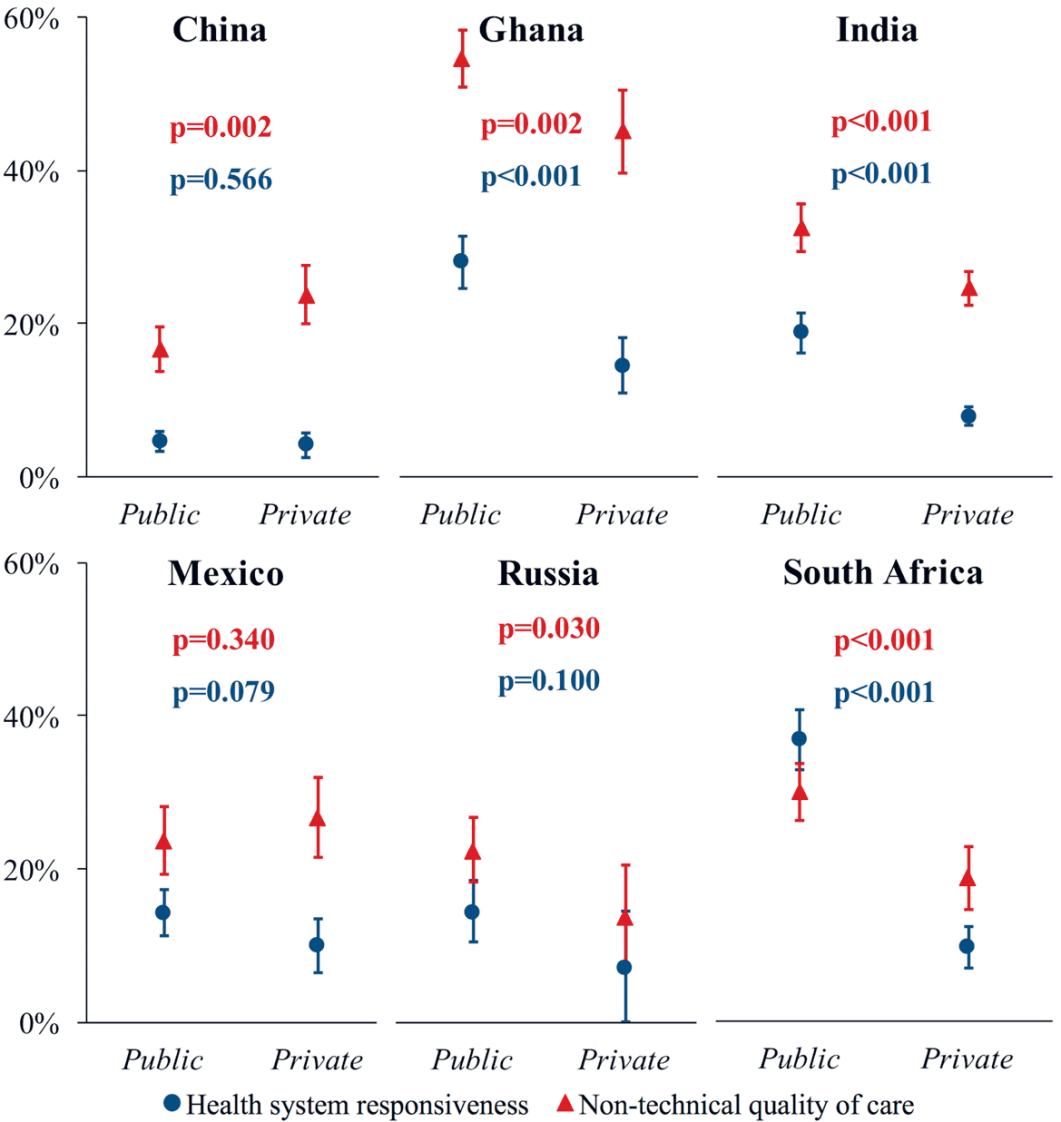
Predicted probability (y axis) of a bad outpatient care rating on at least one dimension, by provider type. For health system responsiveness, a ‘bad’ rating was a rating of “very bad” or “bad” on a five-point Likert scale. For non-technical quality of care, a ‘bad’ rating was a rating of one’s experience for the most recent outpatient visit worse than that described in the vignette scenario. Predicted probabilities were obtained from multivariable logistic regressions with the following co-variates: age (continuous), sex (binary), rural or urban (binary), education (categorical), wealth quintile (categorical), country (categorical), health care provider type (categorical), and whether the household member had health insurance (binary). In addition, the models included an interaction term between each country and provider type. The predicted probabilities for public and private provider were calculated holding other co-variates at their observed values (‘average marginal effects’) as recommended by Hanmer et al [19]. Vertical lines show 95% confidence intervals obtained through the delta method. *P*-values were not adjusted for multiple hypothesis testing.

In India and South Africa, respondents whose last outpatient visit was with a public provider were more likely to report a bad HSR and QoC experience than those whose last visit was with a private provider (**Figure 4**). Table S2 in **Online Supplementary Document(Online Supplementary Document)** shows that in India and South Africa, wealthier individuals were more likely to report seeking their last outpatient care from a private health care provider type (ie, the provider type that furnishes care with a better HSR and QoC). Healthcare provider type may thus be a mediator of the positive association between household wealth and both HSR and QOC in India and South Africa.

## DISCUSSION

We observed a wide degree of variation in the level of HSR and QoC between the six study countries. The proportion of participants who gave a bad rating for their last outpatient care visit on at least one of seven dimensions varied by a factor of 7.7 (from 4.3%, 95% CI = 2.8-6.7, to 33.1%, 95% CI = 23.6-44.2) between countries for HSR, and by a factor of 3.0 (from 17.0%, 95% CI = 11.4-24.5, to 50.8%, 95% CI = 46.0-55.6) for QoC. More generally, we found that the Chinese health system provided (on average across its population) the most responsive care while the health systems in the two African countries (Ghana and South Africa) had the lowest HSR. Ghana’s health system also furnished the worst level of QoC. With regards to the distribution of HSR and QoC between population groups within countries, wealthier individuals in India and South Africa were less likely to report experiencing bad care at their last outpatient visit than poorer participants. The fact that wealthier individuals in these two countries were more likely to seek care from private providers than poorer ones, and that private providers tended to furnish care with a higher HSR and QoC in both countries, may be partially responsible for the wealth gradient in HSR and QoC that we observed in India and South Africa.

Despite its importance to the effective UHC agenda, the existing body of literature on HSR is scant. The first attempt to assess HSR across countries was made by the WHO for the World Health Report 2000 [6]. As no population-based data on HSR were available at the time, HSR was assessed using key informant interviews in 35 different countries (and imputed for other countries). The World Health Surveys included questions on HSR but, to our knowledge, only two studies have examined HSR determinants using this data, which was collected more than a decade ago (in 2002 and 2003) [23,24]. Unlike our study, which analysed differences in HSR between population groups *within* countries, these studies investigated country-level determinants of HSR, such as public health expenditure per capita. Other studies on HSR were conducted at health care facilities among small patient populations: one among mental health care patients in Tehran [25] and in Hanover, [26] patients with diabetes mellitus in Tehran, [27] and among hospitalised patients in Mashhad, Iran [28]. To our knowledge, thus far, only three studies have used population-based data to examine differences in HSR between population groups [29-31] – all of which were restricted to single countries. While the literature on HSR experienced by different population groups is limited, there are several studies on QoC for childbirth in developing countries. For example, a recent mixed-methods systematic review on the mistreatment of women in obstetric services identified 12 qualitative studies (set in Afghanistan, Canada, Cambodia, Ghana, Kenya, Macedonia, Morocco, Serbia, Sierra Leone, South Africa, Tanzania, and the United Kingdom), which reported that women felt they were receiving worse care than their wealthier peers at facilities during childbirth because they were unable to pay service fees or bribes [32]. In addition, four studies – set in rural northern Ghana, rural eastern Tanzania, rural southern Tanzania, and Nairobi (Kenya) – found that women reported being humiliated by health workers for their poverty or lack of education [33-36].

We found that the health systems in India and South Africa provided care with lower HSR and QoC to poorer individuals. This observation could be due to differences between wealth groups in i) which health care facilities they access, and ii) how they are treated by clinicians at the same facilities. While it is difficult to disentangle these pathways in a cross-sectional data set, our findings suggest that utilisation of ‘better’ health care facilities by the wealthy can only partially explain the HSR and QoC gradient by wealth. Specifically, the differences by wealth continue to exist in the regressions after adjusting for provider type, rural vs urban residency, and having health insurance – all variables that are plausibly proxy indicators for utilisation of different health care facilities between respondents. In addition, differences in HSR and QoC between the most and least wealthy quintile were smallest on the cleanliness dimension (a dimension that is reflective of a facility as a whole rather than of the clinician-patient interaction) in both India and South Africa. This study therefore suggests that clinicians at the same facilities may provide less responsive care and care with lower QoC to poorer compared to wealthier patients.

Given the importance of health care quality for effective UHC and the limited evidence base on how to affect HSR and QoC (particularly in low-and middle-income countries), rigorous evaluations of interventions to improve these outcomes are urgently needed. Improving HSR and QoC could involve interventions at the levels of the community, the patient, the clinician, facility management, and the larger health system. For instance, there is some limited evidence that the establishment of community groups and the creation of official community participation mechanisms in local health care delivery can improve aspects of QoC in low and middle-income countries [37]. Similarly, there is some evidence that patient coaching and the provision of relevant educational materials to patients can improve the patient-clinician interaction [38]. While these studies were carried out in high-income settings, coaching of patients on effective behavioural strategies for health care consultations and on how to navigate the health system may also be feasible in less resourced settings. For example, in many low- and middle-income countries, the deployment of lay counsellors and community health workers is common. These cadres could provide information and coaching sessions to patients, such as during community health workers’ home visits. Such sessions might include counselling on how to effectively communicate one’s ideas, concerns, and expectations during a health care consultation process; provision of information on the expectations that the Ministry of Health and other bodies have for how physicians and nurses treat patients at particular health care facilities (including fees for different types of services); information on the various sources of care-seeking that are available in particular settings and when each might be most appropriate; and help with accessing mechanisms for providing feedback on the quality of care and submitting complaints. At the clinician-level, countries may choose to place a greater emphasis on communication skills training for clinicians [39] and provide tools for effectively communicating with patients, especially with those from more deprived socio-economic backgrounds, in medical and nursing school curricula.

This study has several limitations. First, neither the assumption of vignette equivalence nor the assumption of response consistency is verifiable for this data set. However, there is encouraging evidence from the World Health Surveys that the assumption of vignette equivalence holds when using vignette scenarios to adjust for expectations of QoC.[12] Second, the sample for the primary analyses in this study was restricted to those who sought outpatient care in the 12 months preceding the interview. Our sample was thus less likely contain adults in each of the countries’ populations who sought outpatient care infrequently. This issue by itself would not bias our results if i) there is no difference between frequent and infrequent outpatient care attenders in how they perceive to have been treated with regards to HSR and QoC, or ii) one believes that the perceptions of those who seek care more frequently are more important (and should therefore have a higher influence in the measure of the health system’s HSR and QoC) than the perceptions of those who seek care less frequently because the former group is the one who uses the service more regularly. Importantly, because the proportion of SAGE participants who reported to have sought outpatient care in the preceding 12 months varied widely between countries, the validity of the comparison between countries also relies on one of these two assumptions being true. One important scenario that would bias our results is if some participants did not seek care in the preceding 12 months because they were more likely to experience bad HSR or QoC than other participants. For example, some participants may have delayed seeking care because they had experienced bad HSR or QoC in the past, and were thus likely to experience bad HSR or QoC again. Lastly, the SAGE surveys aimed to be nationally representative only among adults aged 50 years and older. Our results should thus be interpreted as primarily applying to this age group only.

## CONCLUSIONS

Pooling data that are nationally representative for adults aged 50 years and older across six large middle-income countries, we found important differences in HSR and QoC between and within countries. Identifying significant disparities in these outcomes by household wealth in India and South Africa, this study highlights the need for the health systems in these countries to improve the HSR and QoC provided to poorer population groups if they are to achieve UHC with good-quality health care services. Of particular concern is that the differences in HSR and QoC by household wealth may also contribute to inequalities in health, by stymieing utilisation of both curative and preventive services among poorer population groups who often need health care the most. This, in turn, may worsen the underlying inequalities in income, education, and wider opportunities that exist in the study countries.
