# Supplementary material for: Non-technical health care quality and health system responsiveness in middle-income countries: a cross-sectional study in China, Ghana, India, Mexico, Russia, and South Africa
Source: J Glob Health. 2018 Oct 15;8(2):020417. doi: 10.7189/jogh.08.020417 (PMC6189548; doi:10.7189/jogh.08.020417)
Supplement: Online Supplementary Document [file jogh-08-020417-s001.pdf]

Online Supplementary Document

Geldsetzer et al. Non-technical health care quality and health system responsiveness in middle-income countries: a cross-sectional study in China, Ghana, India, Mexico, Russia, and South Africa

J Glob Health 2018;8:020417

S1 Text. Questions on health system responsiveness and non-technical quality of care in the SAGE questionnaire 2

S2 Text. Vignette texts in the SAGE questionnaire 3

S1 Figure. Percentage of respondents giving a bad rating when restricting the sample those whose last outpatient care visit was ≤2 months prior to the interview, by country 4

S2 Figure. Percentage of respondents giving a bad rating for their last inpatient care visit, by country 5

S1 Table. Regression results when restricting the sample to those with a recall period of ≤2 months (n=14,103) 6

S2 Table. Multivariable logistic regressions of the last outpatient visit being with a private provider on wealth quintile, by country 8

References: 9

Appendix S1. Questions on health system responsiveness and non-technical quality of care in the SAGE questionnaire

Prompt for the last outpatient care visit:
Now I would like you to think about your most recent visit again. I want to know your impressions of your most recent visit for health care. I would like you to rate your experiences using the following questions.

For your last visit to a health care provider, how would you rate the following:

Prompt for the last inpatient care visit:
Now I want you to think again about your most recent overnight stay. I would like to ask you about your impressions of your last overnight stay. I would like you to rate your experiences using the following questions.

For your last overnight visit to a hospital or longterm care facility, how would you rate the following:

Questions for each dimension used for both the last outpatient and inpatient visit:

	Very good	Good	Moderate	Bad	Very bad
... the amount of time you waited before being attended to?	1	2	3	4	5
...your experience of being treated respectfully?	1	2	3	4	5
…how clearly health care providers explained things to you?	1	2	3	4	5
…your experience of being involved in making decisions for your treatment?	1	2	3	4	5
...the way the health services ensured that you could talk privately to providers?	1	2	3	4	5
...the ease with which you could see a health care provider you were happy with?	1	2	3	4	5
...the cleanliness in the health facility?	1	2	3	4	5

Appendix S2. Vignette texts in the SAGE questionnaire

I am now going to read you stories about people's experiences with health care services. I want you to think about these people's experiences as if they were your own. Once I have finished reading each story, I will ask you to rate what happened in the story as very good, good, moderate, bad or very bad.

[Stan] broke his leg. It took an hour to be driven to the nearest hospital. He was in pain but had to wait an ho operated on the next day.		
How would you rate the amount of time [Stan] waited before being attended to?	Very good	Good
	1	2
[Patricia] went to a crowded clinic. No-one greeted her. She waited for 30 minutes when a nurse called for h screen that separated the waiting area from the examination area.		
How would you rate [Patricia's] experience of being greeted and talked to respectfully?	Very good	Good
	1	2
[Mario] has been told that he has epilepsy and that he needs to take medication. The doctor has very briefly is very busy and there is a queue of patients waiting to see him. Mario would like to know more about what time to ask questions. The doctor says goodbye to Mario, and Mario leave the office.		
How would you rate [Mario's] experience of how clearly health care providers explained things to him?	Very good	Good
	1	2
[José] shared a hospital room with four other persons. There was a toilet for his ward located along the outsi once a week, was occasionally dusty, and had only 1 or 2 chairs for visitors.		
How would you rate the cleanliness of [José's] room inside the facility and provision for toilets?	Very good	Good
	1	2
When the clinic is not busy. [Mamadou] can choose which doctor he sees. But most often it is busy and the		
How would you rate [Mamadou's] freedom to choose his health care provider?	Very good	Good
	1	2
[Alouine] has his consultation behind a screen separating the consultation area from the waiting area. He ha other people hearing his conversation.		
How would you rate the way the health services ensured [Alouine] could talk privately to the health care providers?	Very good	Good
	1	2
[Robert] had a broken arm. The doctor explained different ways of fixing it and then ordered some blood tes needed blood tests and was worried until the doctor explained what they were for.		
How would you rate [Robert's] experience of being involved in making decisions about his health care or treatment?	Very good	Good
	1	2

Figure S1. Percentage of respondents giving a bad rating when restricting the sample those whose last outpatient care visit was ≤2 months prior to the interview, by country[1,2,3]

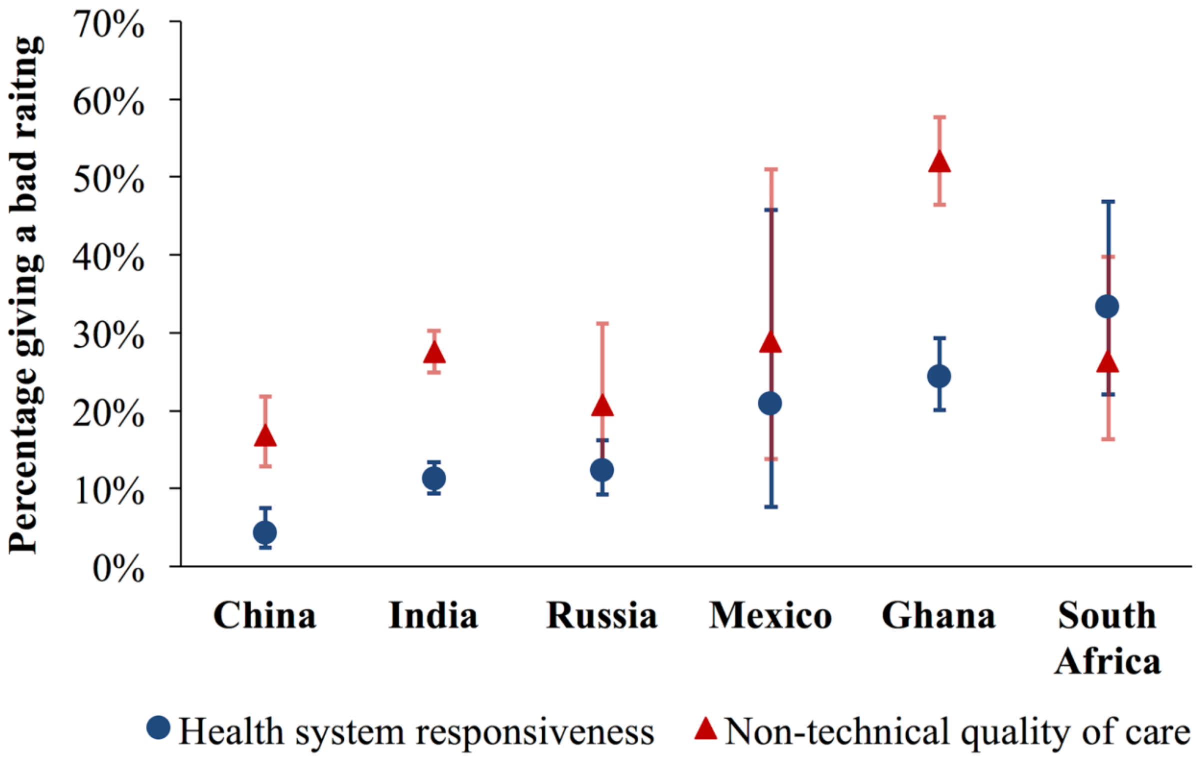

[1] For health system responsiveness, a ‘bad’ rating was a rating of “very bad” or “bad” on a five-point Likert scale. For non-technical quality of care, a ‘bad’ rating was a rating of one’s experience for the most recent outpatient visit worse than that described in the vignette scenario.

[2] Vertical lines show 95% confidence intervals.

[3] Using a Wald test (that follows a F-distribution) for testing the joint significance of ‘country’ as a categorical independent variable in a logistic regression model for survey-weighted data, we rejected (at $\alpha < 0.05$) the null hypothesis that the mean probability of a bad outpatient rating is equal between countries with $p<0.001$ for both outcomes.

Figure S2. Percentage of respondents giving a bad rating for their last inpatient care visit, by country[1,2,3]

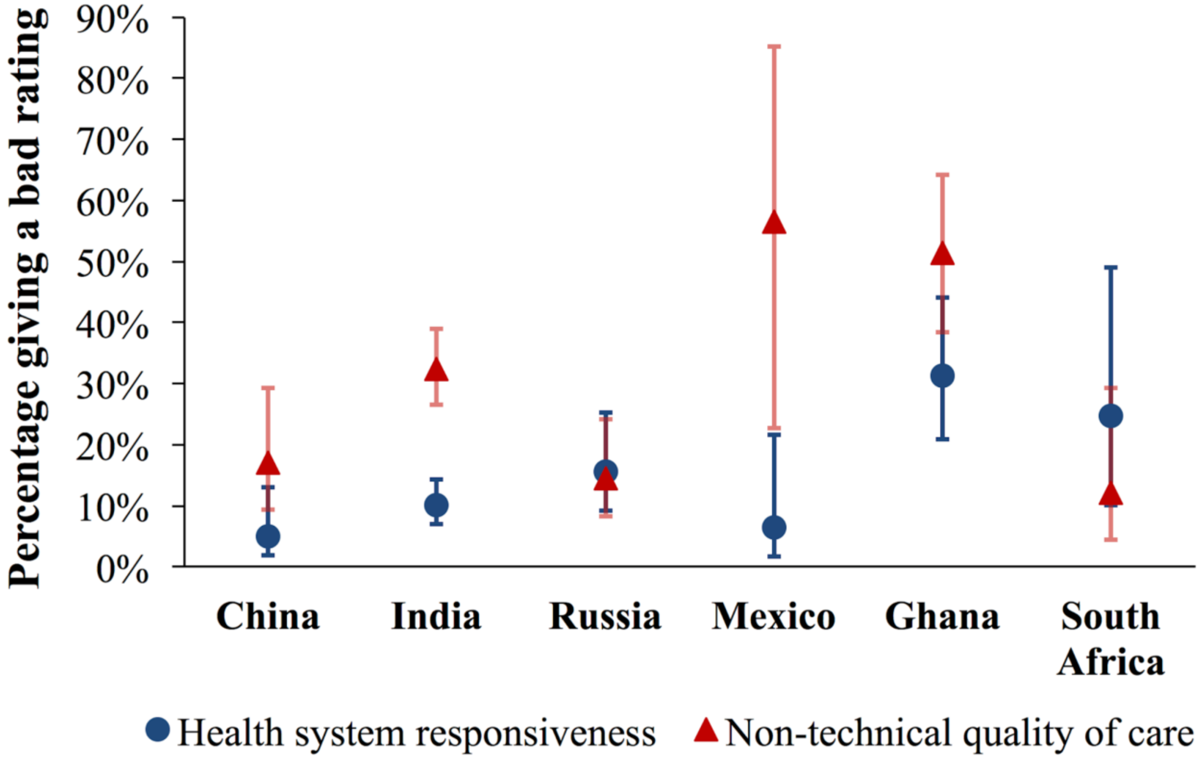

[1] For health system responsiveness, a 'bad' rating was a rating of "very bad" or "bad" on a five-point Likert scale. For non-technical quality of care, a 'bad' rating was a rating of one's experience for the most recent inpatient visit worse than that described in the vignette scenario.
[2] Vertical lines show 95% confidence intervals.
[3] Using a Wald test (that follows a F-distribution) for testing the joint significance of 'country' as a categorical independent variable in a logistic regression model for survey-weighted data, we rejected (at $\alpha < 0.05$) the null hypothesis that the mean probability of a bad inpatient rating is equal between countries with $p<0.001$ for both outcomes.

Table S1. Regression results when restricting the sample to those with a recall period of ≤2 months (n=14,103)[1,2,3]

	Models 1-7				Model 8				Model 9			
	RR	*P*	P^{Holm}	P^{BH}	*RR*	*P*	P^{Holm}	P^{BH}	*RR*	*P*	P^{Holm}	P^{BH}
Outcome: bad health system responsiveness rating[4]												
Household wealth quintile												
1 (poorest)	1.00 (Ref)	-	-	-	1.00 (Ref)	-	-	-	1.00 (Ref)	-	-	-
2	1.02 (0.88 - 1.18)	0.822	1.000	0.930	1.01 (0.87 - 1.17)	0.933	1.000	0.933	1.02 (0.88 - 1.18)	0.832	1.000	0.922
3	0.95 (0.82 - 1.10)	0.480	1.000	0.816	0.93 (0.80 - 1.08)	0.312	1.000	0.727	0.93 (0.81 - 1.08)	0.369	1.000	0.679
4	0.91 (0.78 - 1.06)	0.241	1.000	0.586	0.88 (0.75 - 1.03)	0.102	1.000	0.357	0.88 (0.75 - 1.02)	0.098	1.000	0.278
5 (wealthiest)	0.72 (0.61 - 0.86)	<0.001	0.004	0.001	0.68 (0.57 - 0.82)	<0.001	<0.001	<0.001	0.71 (0.59 - 0.85)	<0.001	0.003	0.001
Education												
No schooling	1.00 (Ref)	-	-	-	1.00 (Ref)	-	-	-	1.00 (Ref)	-	-	-
Some primary school	1.01 (0.88 - 1.14)	0.930	1.000	0.930	1.02 (0.89 - 1.16)	0.763	1.000	0.933	1.02 (0.90 - 1.17)	0.720	1.000	0.922
Completed primary school	0.98 (0.86 - 1.12)	0.735	1.000	0.930	1.01 (0.88 - 1.16)	0.891	1.000	0.933	1.02 (0.89 - 1.17)	0.769	1.000	0.922
Completed secondary school	0.87 (0.74 - 1.02)	0.084	1.000	0.284	0.93 (0.79 - 1.10)	0.389	1.000	0.777	0.96 (0.82 - 1.13)	0.632	1.000	0.922
Completed high school	1.07 (0.93 - 1.24)	0.349	1.000	0.741	1.18 (1.01 - 1.38)	0.039	0.508	0.274	1.22 (1.04 - 1.42)	0.012	0.172	0.052
Completed college or university	0.79 (0.62 - 1.02)	0.067	0.941	0.284	0.93 (0.73 - 1.19)	0.568	1.000	0.795	0.99 (0.77 - 1.27)	0.952	1.000	0.952
Rural	0.96 (0.85 - 1.09)	0.551	1.000	0.852	0.89 (0.78 - 1.01)	0.065	0.775	0.301	0.89 (0.79 - 1.01)	0.079	1.000	0.270
Age group												
< 50 years	1.00 (Ref)	-	-	-	1.00 (Ref)	-	-	-	1.00 (Ref)	-	-	-
50-59 years	0.99 (0.87 - 1.13)	0.930	1.000	0.930	1.01 (0.88 - 1.15)	0.894	1.000	0.933	0.99 (0.87 - 1.13)	0.868	1.000	0.922
60-69 years	1.02 (0.90 - 1.16)	0.731	1.000	0.930	1.04 (0.91 - 1.19)	0.542	1.000	0.795	1.01 (0.89 - 1.16)	0.835	1.000	0.922
≥70 years	0.94 (0.81 - 1.08)	0.397	1.000	0.749	0.95 (0.82 - 1.11)	0.532	1.000	0.795	0.93 (0.80 - 1.08)	0.315	1.000	0.670
Female	1.06 (0.98 - 1.15)	0.158	1.000	0.448	1.05 (0.97 - 1.15)	0.232	1.000	0.650	1.05 (0.97 - 1.14)	0.227	1.000	0.551
Has health insurance	1.01 (0.88 - 1.16)	0.890	1.000	0.930	-	-	-	-	1.07 (0.92 - 1.24)	0.399	1.000	0.679
Provider type							-	-				
Private	1.00 (Ref)	-	-	-	-	-	-	-	1.00 (Ref)	-	-	-
Public	2.24 (1.94 - 2.58)	<0.001	<0.001	<0.001	-	-	-	-	2.20 (1.91 - 2.54)	<0.001	<0.001	<0.001
Other[6]	1.54 (1.30 - 1.82)	<0.001	<0.001	<0.001	-	-	-	-	1.50 (1.26 - 1.78)	<0.001	<0.001	<0.001
Outcome: bad non-technical quality of care rating[5]												
Household wealth quintile												
1 (poorest)	1.00 (Ref)	-	-	-	1.00 (Ref)	-	-	-	1.00 (Ref)	-	-	-
2	0.95 (0.87 - 1.04)	0.246	1.000	0.418	0.96 (0.88 - 1.05)	0.352	1.000	0.704	0.96 (0.88 - 1.04)	0.308	1.000	0.623
3	0.87 (0.80 - 0.96)	0.004	0.042	0.009	0.90 (0.82 - 0.98)	0.019	0.209	0.066	0.89 (0.81 - 0.98)	0.012	0.173	0.053
4	0.79 (0.72 - 0.87)	<0.001	<0.001	<0.001	0.83 (0.75 - 0.91)	<0.001	0.002	0.001	0.81 (0.74 - 0.90)	<0.001	0.001	<0.001
5 (wealthiest)	0.66 (0.59 - 0.73)	<0.001	<0.001	<0.001	0.70 (0.63 - 0.79)	<0.001	<0.001	<0.001	0.69 (0.62 - 0.78)	<0.001	<0.001	<0.001
Education												
No schooling	1.00 (Ref)	-	-	-	1.00 (Ref)	-	-	-	1.00 (Ref)	-	-	-
Some primary school	0.97 (0.89 - 1.06)	0.483	1.000	0.712	0.99 (0.90 - 1.08)	0.761	1.000	0.820	0.99 (0.91 - 1.08)	0.827	1.000	0.879
Completed primary school	0.92 (0.84 - 1.01)	0.084	0.844	0.179	0.97 (0.89 - 1.07)	0.566	1.000	0.720	0.98 (0.89 - 1.07)	0.667	1.000	0.756

Completed secondary school	0.85 (0.77 - 0.94)	0.001	0.017	0.004	0.95 (0.85 - 1.05)	0.289	1.000	0.675	0.95 (0.86 - 1.05)	0.330	1.000	0.623
Completed high school	0.85 (0.77 - 0.94)	0.002	0.028	0.007	0.96 (0.87 - 1.07)	0.505	1.000	0.707	0.97 (0.87 - 1.08)	0.534	1.000	0.720
Completed college or university	0.74 (0.63 - 0.87)	<0.001	0.004	0.001	0.92 (0.78 - 1.07)	0.279	1.000	0.675	0.91 (0.78 - 1.07)	0.277	1.000	0.623
Rural	1.25 (1.13 - 1.38)	<0.001	<0.001	<0.001	1.13 (1.02 - 1.25)	0.015	0.181	0.066	1.14 (1.03 - 1.25)	0.011	0.172	0.053
Age group												
< 50 years	1.00 (Ref)	-	-	-	1.00 (Ref)	-	-	-	1.00 (Ref)	-	-	-
50-59 years	1.03 (0.95 - 1.12)	0.516	1.000	0.712	1.03 (0.95 - 1.12)	0.433	1.000	0.707	1.04 (0.95 - 1.13)	0.410	1.000	0.642
60-69 years	1.01 (0.93 - 1.10)	0.800	1.000	0.886	1.01 (0.92 - 1.10)	0.910	1.000	0.910	1.00 (0.92 - 1.09)	0.962	1.000	0.962
≥70 years	1.00 (0.91 - 1.10)	0.943	1.000	0.943	0.98 (0.88 - 1.08)	0.639	1.000	0.746	0.97 (0.88 - 1.07)	0.566	1.000	0.720
Female	0.99 (0.93 - 1.05)	0.658	1.000	0.799	0.98 (0.92 - 1.04)	0.479	1.000	0.707	0.98 (0.92 - 1.04)	0.415	1.000	0.642
Has health insurance	0.97 (0.89 - 1.06)	0.544	1.000	0.712	-	-	-	-	1.03 (0.94 - 1.12)	0.593	1.000	0.720
Provider type							-	-				
Private	1.00 (Ref)	-	-	-	-	-	-	-	1.00 (Ref)	-	-	-
Public	1.08 (0.98 - 1.18)	0.133	1.000	0.250	-	-	-	-	1.07 (0.98 - 1.17)	0.136	1.000	0.462
Other[6]	0.99 (0.89 - 1.09)	0.834	1.000	0.886	-	-	-	-	0.93 (0.84 - 1.03)	0.174	1.000	0.492

Abbreviations: RR=Risk Ratio; CI=Confidence Interval; Ref=reference level.

[1] These regression models were Poisson regressions with a robust error structure.[1] Standard errors were adjusted for clustering at the level of the primary sampling unit.

[2] Models 1-7 included each of the independent variables shown in the table separately plus country-level fixed effects. Model 8 included household wealth quintile, education, rural versus urban, age group, sex, and country-level fixed effects as independent variables. Model 9 included household wealth quintile, education, rural versus urban, age group, sex, health insurance status, healthcare provider type, and country-level fixed effects as independent variables.

[3] P^{Holm} and P^{BH} refer to p-values that were adjusted for multiple hypothesis testing using the Holm method and the method developed by Benjamini and Hochberg, respectively.[2, 3] Adjustment for multiple hypothesis testing was done separately for the outcome adjusted and unadjusted for vignette response. P-values from models 1-7 were adjusted jointly (i.e., for 17 hypotheses), while p-values from models 8 and 9 were adjusted separately (i.e., 14 hypotheses and 17 hypotheses, respectively).

[4] A 'bad' rating was a rating of "very bad" or "bad" (on a 5-point Likert scale) on at least one of seven health system responsiveness dimensions.

[5] A 'bad' rating was a rating of one's experience for the most recent outpatient visit worse than that of the vignette character on at least one of seven non-technical quality of care dimensions.

[6] This includes charity clinics and hospitals, home visits, "other", and "don't know".

Table S2. Multivariable logistic regressions of the last outpatient visit being with a private provider on wealth quintile, by country[1,2,3]

	**China** *(n=6,379)*		**Ghana** *(n=2,040)*		**India** *(n=7,082)*		**Mexico** *(n=974)*		**Russia** *(n=2,238)*		**South Africa** *(n=1,974)*	
	OR *(95% CI)*	***P***	***OR*** *(95% CI)*	***P***	***OR*** *(95% CI)*	***P***	***OR*** *(95% CI)*	***P***	***OR*** *(95% CI)*	***P***	***OR*** *(95% CI)*	***P***
Household wealth quintile												
1 (poorest)	1.00 (Ref)	-	1.00 (Ref)	-	1.00 (Ref)	-	1.00 (Ref)	-	1.00 (Ref)	-	1.00 (Ref)	-
2	0.99 (0.78, 1.25)	0.919	0.73 (0.47, 1.13)	0.158	1.18 (0.97, 1.44)	0.089	0.78 (0.46, 1.32)	0.356	1.16 (0.49, 2.75)	0.742	1.60 (0.93, 2.76)	0.092
3	0.64 (0.46, 0.88)	0.006	1.13 (0.76, 1.70)	0.542	1.13 (0.91, 1.39)	0.261	1.10 (0.61, 1.99)	0.745	1.28 (0.61, 2.72)	0.512	1.72 (1.01, 2.93)	0.044
4	0.45 (0.31, 0.65)	<0.001	1.01 (0.67, 1.52)	0.965	1.23 (0.97, 1.56)	0.089	1.04 (0.58, 1.86)	0.892	1.40 (0.60, 3.31)	0.438	1.84 (1.08, 3.13)	0.024
5 (wealthiest)	0.23 (0.14, 0.38)	<0.001	1.45 (0.91, 2.30)	0.119	1.32 (1.00, 1.74)	0.052	0.96 (0.55, 1.68)	0.880	1.61 (0.69, 3.75)	0.272	3.68 (2.10, 6.46)	<0.001

Abbreviations: OR=Odds Ratio; CI=confidence interval.

[1] The dependent variable was coded as 1 if the respondent reported that the last outpatient care visit was with a private provider and 0 if it was with a public provider.

[2] These regressions included the following co-variates: age (continuous), sex (binary), rural or urban (binary), wealth quintile (categorical), education (categorical), and whether the household member has health insurance (binary).

[3] Standard errors were clustered at the level of the primary sampling unit.

References:

1. Zou G: **A modified poisson regression approach to prospective studies with binary data**. *American journal of epidemiology* 2004, **159**(7):702-706.
2. Holm S: **A Simple Sequentially Rejective Multiple Test Procedure**. *Scandinavian Journal of Statistics* 1979, **6**(2):65-70.
3. Benjamini Y, Hochberg Y: **Controlling the False Discovery Rate: A Practical and Powerful Approach to Multiple Testing**. *Journal of the Royal Statistical Society Series B (Methodological)* 1995, **57**(1):289-300.
